# Enhancing carrot (*Daucus carota* var. sativa Hoffm.) plant productivity with combined rhizosphere microbial consortium

**DOI:** 10.3389/fmicb.2024.1466300

**Published:** 2024-11-20

**Authors:** Liping Zhu, Peiqiang Zhang, Shunan Ma, Quan Yu, Haibing Wang, Yuexuan Liu, Song Yang, Yanling Chen

**Affiliations:** ^1^Shandong Provincial Key Laboratory of Applied Mycology, School of Life Sciences, College of Resource and Environment Science, Qingdao Agricultural University, Qingdao, Shandong, China; ^2^Postdoctoral Research Station, Rushan Hanwei Bio-Technical & Science CO., LTD., Weihai, Shandong, China

**Keywords:** carrot, phosphorus-solubilization, potassium-solubilization, nitrogen fixation, rhizosphere, microbial consortium, soil property

## Abstract

**Background:**

Plant growth-promoting rhizobacteria (PGPR) are an integral part of agricultural practices due to their roles in promoting plant growth, improving soil conditions, and suppressing diseases. However, researches on the PGPR in the rhizosphere of carrots, an important vegetable crop, is relative limited. Therefore, this study aimed to isolate and characterize PGPR strains from the rhizosphere soil of greenhouse-grown carrots, with a focus on their potential to stimulate carrot growth.

**Methods:**

Through a screening process, 12 high-efficiency phosphorus-solubilizing bacteria, one nitrogen-fixing strain, and two potassium-solubilizing strains were screened. Prominent among these were *Bacillus firmus* MN3 for nitrogen fixation ability, *Acinetobacter pittii* MP41 for phosphate solubilization, and *Bacillus subtilis* PK9 for potassium-solubilization. These strains were used to formulate a combined microbial consortium, N3P41K9, for inoculation and further analysis.

**Results:**

The application of N3P41K9, significantly enhanced carrot growth, with an increase in plant height by 17.1% and root length by 54.5% in a pot experiment, compared to the control group. This treatment also elevated alkaline-hydrolyzable nitrogen levels by 72.4%, available phosphorus by 48.2%, and available potassium by 23.7%. Subsequent field trials confirmed the efficacy of N3P41K9, with a notable 12.5% increase in carrot yields. The N3P41K9 treatment had a minimal disturbance on soil bacterial diversity and abundance, but significantly increased the prevalence of beneficial genera such as *Gemmatimonas* and *Nitrospira*. Genus-level redundancy analysis indicated that the pH and alkali-hydrolyzable nitrogen content were pivotal in shaping the bacterial community composition.

**Discussion:**

The findings of this study highlight the feasibility of combined microbial consortium in promoting carrot growth, increasing yield, and enriching the root environment with beneficial microbes. Furthermore, these results suggest the potential of the N3P41K9 consortium for soil amelioration, offering a promising strategy for sustainable agricultural practices.

## 1 Introduction

Nitrogen (N), phosphorus (P), and potassium (K) are essential macronutrients for the growth and development of higher plants. However, conventional agricultural practices that heavily utilize chemical fertilizers have been linked to environmental challenges, including soil compaction and aquatic eutrophication (Bhardwaj et al., 2014; Rahman and Dunfu, 2018). The efficiency of N fertilizer uptake by plants is relatively low, at 30–40%, with the remainder contributing to soil acidification, reduced organic matter, and the escalation of serious diseases and pests (Taurian et al., 2013; Ahmed et al., 2017; Rahman and Dunfu, 2018). Phosphorus, critical for plant growth and crop yield, is often limited in agricultural ecosystems, with the majority of applied chemical phosphate becoming fixed in the soil, resulting in a P utilization efficiency of only 5–25% (Kochian, 2012; Alori et al., 2017; George et al., 2017; Timofeeva et al., 2022; Pan and Cai, 2023). Excessive P application not only limits plant uptake but also risks the accumulation of residual P in the soil, and the subsequent eutrophication of water bodies (Powers et al., 2016). Potassium, the third most important plant nutrient after N and P, is involved in plant resistance to biotic and abiotic stresses and is required to activate most different enzymes involved in plant processes (Bakki et al., 2024). With only 2% of soil K in a free soluble form, the need for sustainable fertilization practices is acute. These practices should aim to enhance nutrient bioavailability and reduce environmental impact. Soil microorganisms offer a promising avenue for achieving these goals, presenting a critical breakthrough for sustainable agriculture. Abundant and essential to ecosystem functions, soil microbial communities are the driving force behind biogeochemical cycling. The rhizosphere, the dynamic zone surrounding plant roots, is a hub of microbial diversity and complexity. Within this ecosystem, plant growth-promoting rhizobacteria (PGPR), play a pivotal role in soil health, fertility, and the sustainability production of crops (Saha et al., 2008; Sangoquiza-Caiza et al., 2023). PGPR strains enhance plant growth and yield through a variety of direct mechanisms, including the production of phytohormone production, nitrogen fixation, phosphate and potassium-solubilization, siderophores, and ammonia, as well as by indirect pathways such as hyperparasitism, antibiosis, and generation of lytic enzymes (Backer et al., 2018; Basu et al., 2021; Grover et al., 2021; Fiodor et al., 2023). The application of microbial inoculants containing PGPR has been demonstrated to significantly increase crop production by 12–20% (Glick, 2014; Pérez-Jaramillo et al., 2016; Shi et al., 2023), underscoring their potential as a sustainable agricultural strategy.

Phosphorus solubilizing bacteria (PSB), a subset of PGPR, have been identified as pivotal for promoting plant growth and disease resistance (Billah et al., 2019; Kumar et al., 2021; Shultana et al., 2022). Predominantly isolated from the rhizosphere, these strains are metabolically active and capable of transforming insoluble P into bioavailable forms, garnering attention for their role in sustainable agriculture (Hanif et al., 2015; Timofeeva et al., 2022; Wang S. et al., 2022). The solubilization mechanism involve the secretion of organic acids such as malic, lactic, acetic, citric, and succinic, alongside enzymes like phytase and phosphatase, and chelation through siderophores and extracellular polysaccharides (Rodríguez and Vidal, 1999; Hanif et al., 2015; Wang et al., 2017; Liang et al., 2020). Notably, certain PSBs produce plant growth regulators, including indole acetic acid (IAA) and gibberellin (GA), which significantly influence vegetative growth characteristics (Singh et al., 2011; Narendra Babu et al., 2015; Azaroual et al., 2020). The inoculation of PSB, exemplified by *Acinetobacter pittii* gp-1, has markedly improved soybean growth through increased enzymatic activities and phytohormone levels (He and Wan, 2021). Similarly, *Bacillus* sp. LTAD-52, isolated from rapeseed, has demonstrated a substantial yield increase ranging from 21 to 44% under greenhouse conditions (Valetti et al., 2018). Seven endophytic PBS strains from *Cunninghamia lanceolata* have been found to increase plant height, stem diameter, and biomass by 20 to 40% (Chen J. et al., 2021). The function of nitrogen-fixing bacteria (NFB) as a component of microbial fertilizers is influenced by various factors such as crop type, soil moisture, and organic matter content (Shi et al., 2023). While NFB can benefit certain crops, their efficiency is sometimes limited due to low compatibility with host plants, and weak competitive ability against indigenous rhizosphere bacteria. Without an adequate supply of K, plants will have poorly developed roots, grow slowly, and have lower yields (Meena et al., 2014). Potassium solubilizing bacteria (KSB) can solubilize insoluble potassium minerals such as biotite, feldspar, muscovite, vermiculite, smectite, orthoclase, and mica into soluble forms that are available to plants. In this process, the production of organic acid by microorganisms leads to the dissolution of mineral potassium. In addition, KSB can provide beneficial effects by suppressing pathogens, reducing root-knot nematode, and improving soil nutrients and structure (El-Hadad et al., 2011; Meena et al., 2014). Identified KSB, including *Bacillus mucilaginosus*, *B. edaphicus*, *B. circulans*, *Acidithiobacillus ferrooxidans* and *Paenibacillus* spp have shown positive effects on various crops like wheat (Wang et al., 2020), maize, tomato, apple, and so on (Liu et al., 2012; Meena et al., 2014; Feng et al., 2019; Raji and Thangavelu, 2021; Muthuraja and Muthukumar, 2022; Jiao et al., 2024).

Despite these advancements in PGPR research, a disproportionate focus has been placed on cereals like wheat and maize (Nawaz et al., 2020; Kálmán et al., 2023), and legumes such as soybean (Kuzmicheva et al., 2017), with root crops like carrots receiving comparatively less attention. Carrots are recognized for their health benefits, including antioxidants, fiber, and vitamins that may contribute to cancer prevention (Yi et al., 2023), and they also respond positively to mycorrhizal fungi inoculants, which improve their morphological and biochemical traits (Yadav et al., 2021). However, the impact of PGPR on the agronomic and rhizosphere soil characteristics of carrots remains understudied. On the other hand, although PGPR are naturally present in the soil, their populations are often insufficient to compete with other established bacteria in the rhizosphere. Given that successful rhizosphere colonization is a prerequisite for PGPR to exert their effects on plants (Lodeiro, 2015), inoculating with PGPR is a critical step to increase their numbers within the soil, thus optimizing their agricultural benefits. Recent advances have shown that formulations incorporating diverse strains with multiple functions are increasingly recognized for their superior efficiency in improving soil quality and increasing crop yields compared to single-strain microbial fertilizers with limited functions (Beneduzi et al., 2012). This has led to adoption of a strategic approach of selecting multifunctional PGPR strains native to the plant as active components of inoculants, aiming to increase yield potential.

Therefore, in this study, we isolated PGPR strains native to the carrot rhizosphere and evaluated their effects on carrot growth and soil properties. Three dominant PGPR strains with exceptional abilities in P solubilization, N fixation, and K solubilization, were identified and used in single-strain inoculation tests. The study further investigated the effects of single and combined microbial consortia, as well as their integration with chemical fertilizers, on carrot growth parameters and soil enzymatic activities. Additionally, through sequencing analysis, the impact of microbial fertilizers on soil microbial community structure was evaluated. By employing a sustainable strategy based on a combined microbial consortium, this research provides an innovative approach to carrot cultivation, and offers a holistic solution to contemporary agricultural challenges.

## 2 Materials and methods

### 2.1 Site description and sampling

Sampling sites were from carrot rhizosphere soil at Laixi Carrot Science and Technology College, Qingdao Agricultural University, China (E120° 21′, N36° 39′). The soil, characterized as mortar black, exhibited properties of pH 6.99, 7.16 g/kg organic matter, 175.70 mg/kg alkali-hydrolyzable nitrogen (N), 27.75 mg/kg available phosphorus (P), and117.58 mg/kg available potassium (K).

### 2.2 Isolation and screening of PGPR strains with phosphorus solubilizing, nitrogen-fixing, and potassium solubilizing abilities from the rhizosphere of carrot

Productive carrots with adhering soil were transferred into a sterile bench, and then the non-rhizosphere soil was removed by gentle shaking. The rhizosphere soil collected from 10 samples was mixed into a 50 ml sterile tube. 1 g of rhizosphere soil was mixed with 9 ml of sterile water thoroughly for 30 min and serial dilutions of 10^–1^ to 10^–7^ were performed. Subsequently, 100 μL aliquots of each dilution were spread onto plates of Ashby (per 1 L containing 10 g mannitol, 5.0 g CaCO_3_, 0.2 g KH_2_PO_4_, 0.2 g MgSO_4_⋅7H_2_O, 0.2 g NaCl, 0.1 g CaSO_4_⋅2H_2_O and 15 g agar), phosphate-solubilizing medium [per 1 L containing 10 g glucose, 10 g Ca_3_(PO4)_2_, 0.5 g (NH_4_)_2_SO_4_, 0.3 g MgSO_4_⋅7H_2_O, 0.3 g FeSO_4_⋅7H_2_O, 0.3 g KCl, 0.3 g NaCl, 0.03 g MnSO_4_⋅4H_2_O, and 15 g agar], and potassium-solubilizing medium [per 1 L containing 10 g sucrose, 0.5 g yeast extract, 1.0 g (NH_4_)_2_SO_4_, 2.0 g Na_2_HPO_4_, 0.5 g MgSO_4_⋅7H_2_O, 1.0 g CaCO_3_, 1.0 g potassium feldspar powder, and 15 g agar], to isolate nitrogen-fixing, phosphorus-solubilizing, and potassium-solubilizing bacteria (Chen J. et al., 2021; Jiao et al., 2024). These plates were incubated at 28°C for 3 days and the relative bacterial colonies were selected based on the appearance of hyaline circles (Valetti et al., 2018). The isolates were subjected to successive purification steps and the purified strain was then stored in glycerol stock at −80°C. The solubility index (SI) was determined by measuring hyaline circles and colony diameters at 4 days of culture according to the following formula: SI = (colony diameter + halo diameter)/(colony diameter) (Ibáñez et al., 2021).

### 2.3 Evaluation of the growth-promoting properties of the PGPR strains

#### 2.3.1 Phosphate solubility assay

The phosphate solubility assay was conducted in quadruplicate to ensure accuracy. PSB strains with higher SI values were subjected to further testing in the NBRIP liquid medium (Hanane et al., 2008), which incorporated tricalcium phosphate as the sole source of phosphorus. Inoculations were made into 500 mL Erlenmeyer flasks containing 100 mL of the NBRIP liquid medium, achieving a final concentration of 5 × 10^4^ colony-forming units per mL (CFU/mL) for each bacterial strain. The cultures were incubated at 30°C and 150 rpm for 8 days, with the soluble P content being measured at every 48 h interval as follows: a small volume of the fermentation broth was centrifuged at 10,000 rpm/min for 10 min and the supernatant was collected and diluted if necessary; the reaction mixture was prepared by sequentially adding 1 mL Mo Sb antibody solution and 0.5 mL of ascorbic acid solution, followed by thorough mixing and a 15-min incubation at room temperature. The *P* concentration was measured using the molybdate blue method, with P-free samples serving as blank controls in this study (Jiao et al., 2024).

#### 2.3.2 Determination of nitrogen fixation efficiency

The screened NFB strain was incubated in a 150 mL conical flask with 50 ml of liquid Ashby medium for 7 days at 28°C. The culture was centrifuged (10,000 r/min) for 20 min, and the supernatant was taken for N concentration detection using the micro-Kjeldahl method (Shi et al., 2023). The test was repeated three times with the uninoculated medium as a control.

#### 2.3.3 Determination of potassium solubility

The screened KSB stains were incubated in 50 mL of K solubilizing medium at 28°C with shaking at 180 rpm for 5 days. After incubation, the fermentation broth was centrifuged at 500 rpm for 10 min to remove the insoluble particles. The supernatant was then collected by centrifugation at 10,000 r/min for 10 min. This process was conducted in triplicate, with a control group using an uninoculated medium. Then flame spectrophotometry was used to calculate the effective K content of the supernatant (Jiao et al., 2024).

### 2.4 Strain identification and phylogenetic analysis

The bacteria strains were chosen for molecular identification based on full 16S rRNA sequence, using universal primers, 27F (5′-AGAGTTTGATCCTGGCTCAG-3′) and 1492R (5′-TACGACTTAACCCCAATCGC-3′). The sequencing results were aligned against 16S rDNA homologous sequences in the NCBI database and a high percentage of sequence identity (usually > 97%) is considered indicative of the same species. Subsequently, a phylogenetic tree was constructed using MEGA 6 software and iTOL network (Tamura et al., 2007).^1^

### 2.5 Verification of screened strains on the growth of carrot

#### 2.5.1 Potted experiment design

The experimental procedures were conducted with slight modifications to the method previously described (He and Wan, 2021). Carrot seeds of full viability were selected and subjected to surface disinfection, followed by a soak in a 1% sodium hypochlorite for 5 min, and then thoroughly rinsed with sterile water at least 4 times to remove the disinfectant. These seeds were then placed on two layers of moistened filter paper and incubated at 28°C in the dark for 48 h to germinate. The germinated seeds were sown in sterilized potting soil within containers measuring 20 cm × 20 cm × 15 cm, with each pot receiving 2 kg of soil and 10 seeds, and the seed surface was covered with a thin layer of about 1 cm of soil.

Meanwhile, bacterial strains were inoculated into 250 mL Erlenmeyer flasks containing 100 mL of LB liquid medium and cultured at 25°C, 200 rpm for 48 h. The bacterial cells were harvested by centrifugation at 8,000 *g* for 10 min at room temperature. The pelleted cells were resuspended with sterile water to achieve a bacterial density of 3 × 10^7^ colony-forming units (CFU/mL), which was used for inoculation purposes. Subsequently, two treatments were established: (1) CK, where plants were inoculated with 50 mL of sterile water serving as a control group; (2) the bacterial strain treatment, where plants were treated with 50 ml of bacterial inoculant with 3 × 10^7^ CFU/mL. Each treatment was conducted in triplicate. In addition, the combined microbial consortium and commercial biofertilizer were also treated for the pot experiment, designated as groups N3P41K9, WZF, BSW, and PGA. The N3P41K9 treatment featured a combined microbial consortium comprising MN3, M6P41, and PK9. The WZF, BSW, and PGA treatments involved the application of commercially available microbial biofertilizers. WZF for Wuzhoufeng, contains *Trichoderma harzianum*, *B. subtilis*, and *Paenibacillus* spp, with a product specification of 25 kg/bag, and an effective viable count of at least 200 million/g, purchased by Wuzhoufeng Agricultural Science and Technology Co., LTD.; BSW, for Baisiwang, contains *B. subtilis* and *Bacillus thuringiensis*, comes in 500 g/bag with an effective viable count of at least 1 × 10^10^ CFU/g/g, containing, purchased from Shandong Shigaode Plant Nutrition Technology Co., LTD.; PGA, for Pea-based polyglutamic-acid enhanced microbial agent, is a package in 40 kg/bag with an effective viable count exceeding 5 × 10^8^ CFU/g, containing soybean meal, saprophytic fungi, nitrogen-fixing bacteria, phosphorus-solubilizing bacteria, and potassium-solubilizing bacteria and purchased from Shandong Muyu Shi Biotechnology Co., LTD. Each treatment was standardized to an effective viable count of 200 million/g.

The potted plants were placed in a greenhouse with a photoperiod of 16 h light/8 h darkness and a temperature regime of 25°C/18°C. The soil moisture was meticulously maintained at 40% following the emergence of the carrot seedings. Growth parameters including plant height, fresh weight, root length and root diameter were recorded at 20, 30, and 40 days post-emergence. After 40 days, soil samples were collected from the pots for analysis of available element contents and enzymatic activities.

#### 2.5.2 Field test of combined microbial consortium

This research was conducted in Houtun Village, Dianbu Town, West City, Qingdao City, Shandong Province, China (E120°21’, N36°39’), from July to December 2023. The study region, characterized by a temperate semi-humid monsoon climate, has an average annual temperature of 11.3 °C and receives a mean annual rainfall of 732.5 mm. The test soil used was Shajiang black soil, which has been part of a long-term crop rotation of carrots. The carrot cultivar selected for testing was Outehong 2. The experimental design included ten treatments, each with equal N application and viable bacteria groups: a control (CK) without fertilizer, a complex microbial consortium (N3P41K9), and commercially available microbial biofertilizers (WZF, BSW, PGA), as well as an optimized treatment (OPT: with N 274.5 kg/ha, P_2_O_5_ 49.5 kg/ha, K_2_O 315 kg/ha) combined with each of the microbial agents. Each treatment was replicated three times in a completely randomized layout, with an area of 25 m^2^ per replicate. Each treatment group’s effective viable bacteria count was 2 × 10^8^ CFU/mL. Fertilization of microbial agents was applied before sowing, followed by three additional fertilizer applications at the seedling establishment, the rosette development, and the early phase of fleshy root expansion, all after sampling for measurement. Finally, samples were taken at the seedling emergence stage (Day 20), the rosette stage (Day 40), the expansion stage (Day 80), and the harvest stage (Day 100) for the determination of carrot growth indicators and soil characteristics.

#### 2.5.3 Analysis of plant biomass

Plant biomass was assessed through the collection and processing of carrot samples. The sampling was conducted at the germination stage, rosette stage, fleshy root expansion stage, and harvest stage, to determine plant height, fresh weight, root length, respectively.

#### 2.5.4 Determination of soil properties

In the pot experiment, 20 g of soil surrounding the main roots of the carrots after 40 days of growth was collected, sieved through a 2-millimeter mesh, and then used for the analysis of soil properties. In the field, soil sampling was carried out at the four stages mentioned above, collecting surface soil samples (0–20 cm depth). Three replicates were gathered and mixed to create a composition sol sample. Soil physicochemical properties were determined using standard methods (Bao, 2000). A brief procedure involved the placement of an air-dried soil sample into a triangular flask, followed by the addition of distilled water at a soil-to-water ratio of 1:2.5. The mixture was then incubated at 160 rpm for 2 h, centrifuged, and the pH value of the supernatant was measured. The alkaline-hydrolyzable N was ascertained using the diffusion method (DB13/T 843–2007); the available P level was determined by the molybdenum-antimony colorimetry method (NY/T1121.7–2014); and the available K content was determined by ammonium acetate extraction method (NYT 889–2004). By methodology for soil enzyme research (Miao et al., 2019; Zhang et al., 2023), soil sucrase activity was evaluated using the 3,5-dinitrosalicylic acid colorimetry method, with results expressed as milligrams of glucose after 24 h. Similarly, soil urease activity was measured by the sodium phenoxide colorimetry method, expressed as milligrams of NH_3_-N per gram of soil over 24 h. The final calculation of the change is to indicate the difference, which is determined by subtracting the relevant value of the original soil from the value of the measured sample. All data was recorded after three repetitions of each test.

### 2.6 Analysis of soil microbial community structure

To investigate the impact of the inoculated microbial consortium on indigenous soil microbial communities, fresh soil samples after harvesting were extracted for microbial DNA using a Fast DNA SPIN kit (MP Biomedicals, USA). PCR amplification was performed for bacterial 16S rDNA using primer pairs 515F (5′-GTGCCAGCMGCCGCGG-3′) and 907R (5′-CCGTCAATTCTTTTRAGTTT-3′). Sequencing was conducted on the Illumina MiSeq platform (Beijing Nuohe Zhiyuan Technology Co., Ltd.). Operational taxonomic units (OTUs) clustering was performed using USEARCH (version 10.0) at a similarity level of 97% and filtered using a threshold of 0.005% of the total number of sequences (Bokulich et al., 2013).

### 2.7 Statistical analysis

All data were analyzed by analysis of variance (ANOVA) using SPSS (version 26.0, IBM, Inc.) and expressed as means ( ± SE; standard error), and the plotting was conducted using GraphPad Prism 8.0. The statistically significant level was set as *P* < 0.05, and the extremely significant level was set as *P* < 0.01. In this study, only results with a *P* < 0.05 are reported, while those that are not statistically significant are omitted. Venn diagram and non-metric multidimensional scaling (NMDS) plot were used to reflect bacterial community composition. Pairwise analysis of similarity (ANOSIM) was applied to quantitatively evaluate differences in bacterial community composition. The Shannon index of bacterial community composition was analyzed. An abundance plot of soil microbial communities featuring the species with higher rankings in relative abundance at the phylum and genus levels was created based on the classification and proportional count of OTUs. Pearson correlation analysis was used to study the relationship between plant physiological indexes, soil physical and chemical properties, enzyme activities, and soil microorganisms. Redundancy analysis (RDA) to evaluate the relationship between soil properties and the microbial communities, was performed using the online tool at www.bioincloud.tech/standalone-task-ui/rda. MEGA 6.0 software was applied for phylogenetic trees, employing the Neighbor-Joining method with a Bootstrap value of 1,000.

## 3 Results

### 3.1 Isolation and evaluation of PBS, NFB and KSB strains from the rhizosphere of carrot

From the carrot rhizosphere, a variety of bacteria were isolated, and upon verification considering their growth rates, 15 distinct morphological strains were identified, including 12 PBS, one NFB, and two KSB, as illustrated in Supplementary Figure 1a. The phylogenetic tree constructed from 16S rRNA sequence indicated that these 12 PSB isolates belonged to three genera (two for *Bacillus*, three for *Pseudomonas*, and seven for *Acinetobacter*); the NFB strain MN3 was identified as *Bacillus firmus*; two KSB strains PK9 and MK31 were identified as *B. subtilis* and *Paenibacillus polymyxa*, respectively (Supplementary Figure 1b and Supplementary Table 1).

**FIGURE 1:**
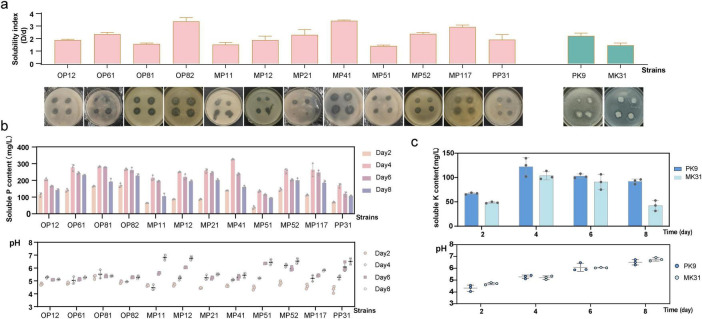
Evaluation of the PSB and KSB strains. **(a)** Soluble index (SI) performance of the strains; **(b)** Phosphorus solubilizing effect and pH analysis of PSB strains in liquid media; **(c)** Potassium solubilizing effect and pH analysis of KSB strains in liquid media.

As seen in Figure 1a, among the 12 PSB strains, OP82 (*B. subtilis*), MP41 (*A. pittii*), and MP117 (*B. velezensis*) displayed the most pronounced hydrolysis halo according to the solubilizing index (SI). The PK9 (*B. subtilis*) showed a higher K SI. Further detection revealed that during the liquid phosphorus fermentation process, the pH value of the medium (with an original pH of 6.8) exhibited an overall trend of “initial decline followed by a gradual rise” in correlation with the duration of phosphorus-solubilization. A marked decrease in pH was observed for most of the strains on Day 2, which was followed by an incline. The highest level of P soluble content was reached on Day 4, with values ranging from 136 to 330 mg/L (Figure 1b), reflecting a notable variance in the solubilization capabilities of the strains. The most effective strains in solubilizing P were MP41, OP61 (*A. pittii*), OP81 (*A. pittii*), OP82, and MP117, and Linear regression analysis between pH and phosphate soluble content revealed a significant negative correlation over the cultivation period (Supplementary Figure 2). Similarly, two KSB strains also showed hydrolytic halos on the medium, with strain PK9 having a higher SI than MK31 (Figure 1a). During the liquid medium cultivation (Figure 1c), the pH significantly decreased on Day 2 from the initial neutrality to around 4.5. The maximum soluble K content also occurred on Day 4, with the strain PK9 at 123.3 mg/L and MK31 at 103.9 mg/L, respectively. It is speculated that phosphate-solubilizing or the potassium-solubilizing ability was related to acid production. However, as the strain metabolizes, the concentration of phosphorus or potassium in the culture decreases and the pH value gradually returns. In addition, after 15 days of cultivation of the NFB strain MN3, the total nitrogen content was measured to reach 21.91 mg/L.

### 3.2 Effect of different strains on the carrot growth and soil-related elements in the pot experiment

**FIGURE 2:**
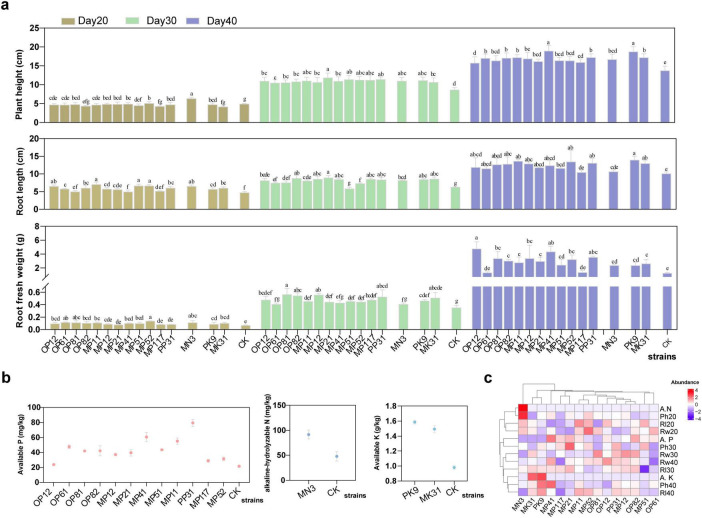
Effect of individual strain on the pot experiment’s carrot growth and soil nutrients. **(a)** Growth metrics for carrots, including plant height, root length, and root fresh weight were recorded on day 20, 30, 40, respectively. Different lowercase letters indicate significant differences (*P* < 0.05) among treatments of different strains. CK represents the sample without inoculation. **(b)** Soil nutrient levels, the content of available phosphorus (P), potassium (K) and alkaline-hydrolyzable nitrogen (N) in the soil on Day 40. **(c)** A heatmap analysis of the impact of each strain on these parameters.

To elucidate the impact of these bacteria strains on carrot growth, we carried out the pot experiment and detected the relative growth indicators of carrots after 20, 30 and 40 days of treatment with different bacterial agents, respectively. As shown in Figure 2a and Supplementary Table 2, plant height, plant fresh weight and root length of most of the treatment groups were significantly higher than the control group. In the early stages of carrot growth (the first 20 days), these strains showed a certain growth-promoting effect, with an average increase in plant height, root length and root fresh weight of 15.5 ± 12.2, 28.4 ± 13.7, and 45.1 ± 23.4%, respectively, compared to the control. Among all the strains, the NFB strain MN3 had the most significant effect, increasing the plant height, root length, and root fresh weight by 54.2 ± 8.9, 39.7 ± 6.8, and 61.6 ± 39.8%, respectively. On Day 30, the growth-promoting effects of the PSB and KSB were more pronounced, such as MP21, OP81, OP82, PP31, PK9, and MK3. However, The PSB strain PK9 had a promoting effect on the plant height, while MK31 had a more obvious promoting effect on the root. On Day 40, the carrot roots began to swell and turn orange, and the growth-promoting effect of the strains was more significant. Among them, MP41, and PK9 showed an advantage in plant height, increasing by 58.4 ± 11.3%, and 56.7 ± 10.7% compared to the control, respectively; OP12, MP41, and PP31 displayed a marked advantage in root fresh weight, increasing by 391.1 ± 101.7, 348.6 ± 78.2, and 267.1 ± 11.5% compared to control, respectively. The NFB strain MN3 also showed a stable growth-promoting effect, increasing plant height, root length, and root fresh weight by 5.2 ± 8.6, 14.8 ± 4.8, and 144.2 ± 8.4%, respectively. OP61 and OP117 were the least significant in promoting growth in all aspects.

Subsequently, the result of the changes in the content of corresponding elements in the soil after Day 40 was illustrated in Figure 2b. The soil’s available P content varied significantly with PP31 resulting in the highest level, followed by MP41. In contrast, strains OP12, MP117, and MP52 had the lowest available P content. Regarding the soil’s available K content, PK9 was 6.3 ± 1.7% higher than MK31. Compared to the control group, MN3 significantly increased the soil’s alkaline-hydrolyzable N content by 94.9 ± 2.7%. Further analysis of growth indicators using the correlation heatmap (Figure 2c) revealed that NFB, as well as PSB and KSB, significantly influenced plant growth at 20, 30, and 40 days post-application of the bacterial agents, respectively. Within the group of PSB, strain MP41 showed a synergistic effect, closely clustered with MN3, PK9, and MK31. Considering the slight superiority of PK9 over MK31, we constructed a microbial consortium N3P41K9 comprising MN3, MP41, and PK9 to assess their collective impact on carrot growth. Additionally, the growth of these three strains did not exhibit any antagonism or inhibition (Supplementary Figure 3).

### 3.3 Effects of microbial consortium N3P41K9 in the pot experiment

**FIGURE 3:**
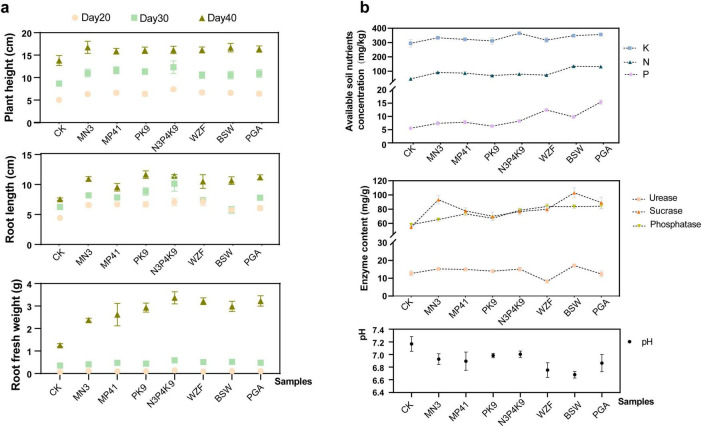
Effect of combined microbial consortium N3P41K9 on the pot experiment’s carrot growth and soil nutrients. **(a)** Growth metrics for carrots, including plant height, root length, and root fresh weight were recorded on Day 20, 30, 40, respectively. **(b)** Soil nutrient levels, including the content of available P, K, and alkaline-hydrolyzable N, as well as the enzyme contents and the soil pH on Day 40. CK represents the blank control without inoculation. The sample inoculated with a single strain of MN3/MP41/PK9 was used as the negative control, while the samples inoculated with commercial fertilizers, like WZF, BSW, and PGA, were used as the positive control.

To thoroughly evaluate the impact of the microbial consortium N3P41K9 on carrot growth, we executed a comparative pot fertilization treatment using commercially available microbial fertilizers WZF, BSW and PGA, as positive controls and single strains as negative controls (Figure 3a and Supplementary Table 3). During the initial phase (Day 20 post-inoculation), no significant difference in carrot growth was detected among the various microbial treatments, while a discernible enhancement in both plant height and root length was noted in comparison to the untreated control. By Day 30, significant differences emerged, with the N3P41K9 group exhibiting the most remarkable increases in plant height (41.8 ± 15.9%) and root length (61.7 ± 19.9%) over the untreated control. The strain MP41 followed, enhancing plant height by 33.7 ± 9.0%, and the strain PK9 notably increased root length by 40.9 ± 9.8%. The impact of MN3, WZF, and PGA microbial agents was comparable, in contrast to BSW, which had a minimal effect. These results suggested that phosphorus-solubilizing bacteria potentially foster root elongation. By Day 40, the microbial treatment showed a discernible impact, with carrot plant height increasing by 17.0 ± 4.8 to 20.7 ± 6.8% and root fresh weight escalating substantially from 137.0 ± 17.8 to 156.1 ± 18.4%. Notably, the consortium N3P41K9 matched the efficacy of commercial microbial agent products. Upon soil sampling and physicochemical analysis after Day 40, it was observed that the application of N3P41K9 resulted in a significant increase in available K by 23.7 ± 2.4%. However, its impact on the availability of P and alkaline-hydrolyzable N in soil was less pronounced compared to commercial microbial products, particularly PGA (Figure 3b and Supplementary Table 3). The PGA, containing multiple strains known for their abilities in P and K solubilization and N fixation, showed a notable increase in available P by 117.9 ± 14.4% and in available K by 20.8 ± 2.7%. Significant increases in phosphatase (from 12.3 ± 3.7 to 44.6 ± 6.6%) and sucrase activity (from 28.3 ± 10.6 to 88.5 ± 12.9%) were observed compared to the control (Figure 3b). The WZF, BSW and PGA groups exhibited the highest phosphatase activity, followed by the N3P41K9 and MP41. In contrast, single strain MN3 and the microbial agent BSW substantially increased sucrase activity by 70.6 ± 10.9 and 88.5 ± 12.9%, respectively. The urease content was increased in all treatment groups compared to the control, except for the WZF. Among them, BSW showed the highest increase, followed by the N3P41K9.

Collectively, these results demonstrated that the microbial consortium N3P41K9 was not only more effective in promoting early carrot growth than individual strains, but also rivals the performance of commercial microbial fertilizers. In terms of soil properties, the consortium N3P41K9 was comparable to the commercial microbial agent PGA, and slightly lower than BSW. The impact of the microbial consortium still requires further validation through field experiments.

### 3.4 Effects of microbial consortium N3P41K9 in the field experiments

**FIGURE 4:**
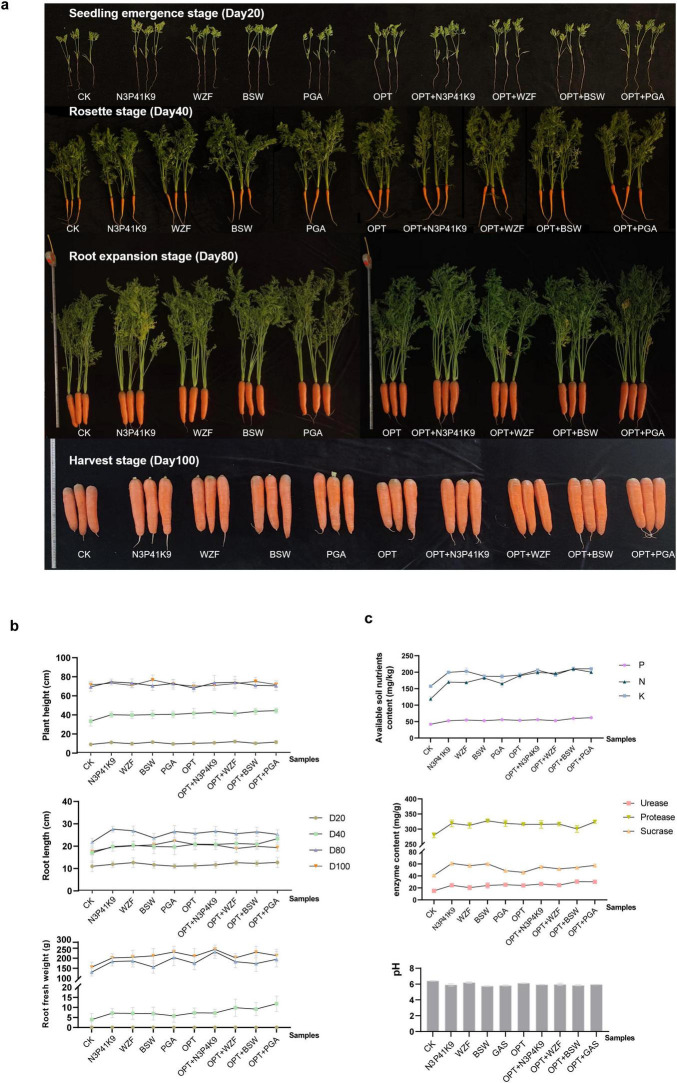
Effect of combined microbial consortium N3P41K9 on the carrot growth and soil nutrients in the field trial. **(a)** Growth morphology of the carrots at different stages under different treatments. **(b)** Growth metrics for carrots, including plant height, root length, and root fresh weight were recorded on Day 20, 40, 80, 100, respectively. **(c)** Soil nutrient levels, including the content of available P, K and alkaline-hydrolyzable N, as well as the enzyme contents and the soil pH on Day 100. CK represents the blank control without inoculation. OPT stands for optimized chemical fertilizer regime used by the farmers. The samples inoculated with commercial fertilizers, like WZF, BSW, and PGA, were used as the positive control.

Furthermore, we validated the effect of the combined microbial consortium N3P41K9 on carrot growth in field trials compared to commercially available microbial agents, WZF, BSW, and PGA. Concurrently, we investigated the efficacy of co-applying microbial agents in conjunction with an optimized chemical fertilizer regimen, designated as OPT, which was customized by farmers and the treatments were categorized into groups: OPT+ N3P41K9, OPT+WZF, OPT+BSW, OPT+PGA. After fertilizing, the carrot growth indicators, and post-harvest soil physicochemical properties were assessed at four distinct developmental stages: the seedling emergence stage (Day 20), the rosette stage (Day 40), the expansion stage (Day 80), and the harvest stage (Day 100). Figure 4a reflects the growth status of carrots under different treatments at various sampling time points. The growth indicators measured at different stages (Figure 4b and Supplementary Table 4) indicated that the exclusive application of microbial agents significantly enhanced carrot plant height by 2.7 ± 2.8 to 20.3 ± 8.9%, root length by 8.1 ± 12.7 to 26.7 ± 6.0%, and fresh root weight by 3.7 ± 15.2 to 82.2 ± 55.4%. The co-application of chemical fertilizers and microbial agents yielded even more pronounced results, with an increase in fresh root weight ranging from 6.1 ± 13.2 to 151.7 ± 109.4%. Notably, the standalone use of N3P41K9 showed a significant advantage in promoting root elongation; in terms of fresh root weight, its performance was comparable to that of WZF but did not match up to PGA. The combined application of N3P41K9 with OPT exhibited a significant advantage in both root length and fresh weight, exceeding the combination of OPT + WZF. In comparison to the control, the application of microbial fertilizer led to a significant increase in the levels of available P and K, and alkaline-hydrolyzable N, with N3P41K9 showing increases of 25.2 ± 5.2, 27.0 ± 1.5, and 43.2 ± 4.5%, respectively (Figure 4c). The use of N3P41K9 also enhanced the soil’s urease, sucrase, and phosphatase content, with increases of 61.3 ± 17.4, 48.9 ± 4.9, and 14.0 ± 4.0%, respectively, without causing a significant drop in soil pH. It is noteworthy that the N3P41K9 treatment outperformed the WZF treatment in terms of alkaline-hydrolyzable N, urease, sucrase, and phosphatase content. These results suggest that the composite microbial consortium developed in this study is competitive with the commercially available microbial agent WZF, slightly less effective than PGA, but markedly superior to BSW.

### 3.5 Effect of combined microbial consortium N3P41K9 on soil bacterial community

**FIGURE 5:**
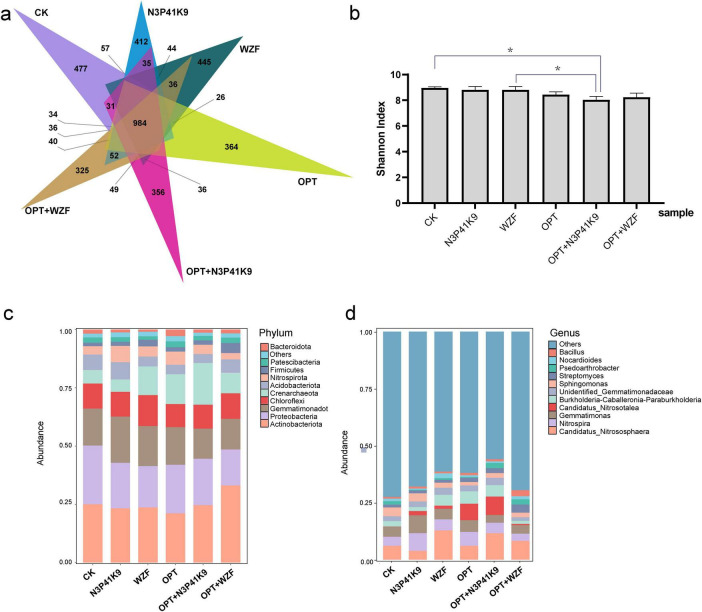
Composition of rhizosphere microbial community. **(a)** The Venn diagram shows the unique and shared core microbiomes among six groups. **(b)** Shannon index of the microbial community of different groups. Asterisks denote significance (*p < 0.05). The stacking diagram reflects the impact of the microbial fertilizers on the relative abundance of the bacterial communities in the rhizosphere soils, at the phylum level **(c)** and the genus level **(d)**. The top 10 bacterial phyla and top 11 bacterial genera with relative abundance > 1% are displayed.

The diversity and composition of the soil bacterial communities from the treatment of consortium N3P41K9 were collected after the harvest of carrots and analyzed based on high-throughput sequencing. It showed that whether it was the application of microbial agents, chemical fertilizers, or the combined use of both, there was a certain impact and fluctuation on the structure and quantity of the soil microbial community. A total of 5,449 OTUs were found across all the samples, and they shared 984 OTUs (Figure 5a). The NMDS result showed a distinct difference in bacterial community composition among the six groups with a stress of 0.0518 (Supplementary Figure 4). The N3P41K9 group showed the closest relation with the CK group and OPT+WZF group. The chemical fertilizer-used groups (OPT, OPT+N3P41K9, OPT+WZF) were separated from the other microbial fertilizer-used groups on the MDS1 dimension. Besides, The WZF and OPT groups are most closely related on the MDS2 dimension. ANOSIM confirmed further the significant difference (*R* = 0.65, *p* = 0.001). The bacterial community diversity represented by the Shannon indexes was significantly lower in fertilizer-used groups than in other treatments (Figure 5b).

At the phylum level (Figure 5c), compared to the control group CK, all the treatment groups mainly altered the proportions of the Actinobacteriota, Gemmatimonadota, Chloroflexi, Crenarchaeota, and Nitrospirota. Among them, the group using only chemical fertilizers (OPT) significantly increased the proportion of Crenarchaeota. The application of the microbial agent N3P41K9 increased the proportion of Gemmatimonadota, while the WZF group mainly increased the proportion of Crenarchaeota and decreased the proportion of Proteobacteria. The combination of chemical fertilizer and microbial agents, OPT+N3P41K9, significantly increased Crenarchaeota, while OPT+WZF significantly increased the proportion of Actinobacteriota. At the genus level (Figure 5d), compared to the control group CK, all treatment groups increased the genus *Candidatus* Nitrosotalea. In addition, a single use of N3P41K9 was able to increase the proportions of *Nitrospira* and *Gemmatimonas*, and a single use of WZF had a significant impact on the soil, notably increasing the proportion of Candidatus Nitrososphaera. The application of the chemical fertilizer OPT significantly led to an increase in *Candidatus* Nitrosotalea in the soil. Compared with the control OPT, the combination of OPT+ N3P41K9 mainly caused an increase in *Candidatus* Nitrososphaera, while OPT+WZF showed a significant difference in the impact on soil microbial communities. It can be seen that the application of microbial agents alone had the least overall impact on the balance of soil microbial communities. Particularly, N3P41K9 showed a relatively stable characteristic when used alone or in combination with chemical fertilizers, resulting in minimal fluctuations in the soil microbial community. However, WZF caused greater microbial community fluctuations, especially when used in combination with chemical fertilizers.

### 3.6 Relationships between soil properties and microbial communities

**FIGURE 6:**
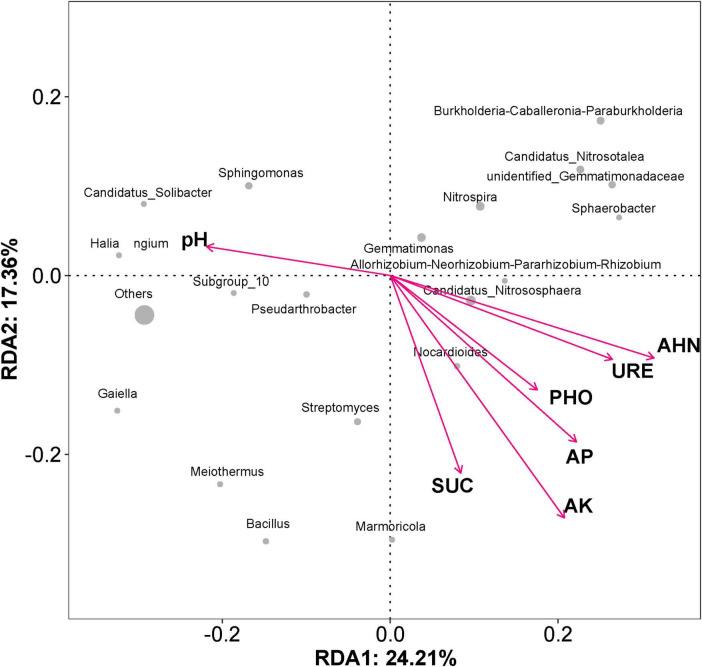
Redundancy analysis of the impacts of soil properties on rhizosphere bacterial community structure. The arrow length represents the intensity of the indicated environmental factor’s influence on community changes. The angle between the arrow and the axis represents the correlation between the indicated environmental factor and the axis; smaller angles correspond to higher correlations. The distance between each sample point and arrow indicates the strength of the effect of that environmental factor on the sample. The location of a sample in the same direction as an arrow indicates a positive correlation between the environmental factor and changes in the microbial community; conversely, alignment in the opposite direction indicates a negative correlation. AP, available phosphorus; AK, available potassium; AHN, alkaline-hydrolyzable nitrogen; URE, urease; SUC, sucrase; PHO, phosphatase. The gray dots represent the abundance of the dominant genera.

The structure and composition of the soil microbial community are closely linked to soil’s physical and chemical properties. Redundancy analysis (RDA) (Figure 6) showed that the soil properties had a significant impact on the soil microbial community structure. Environmental factors on the first ordination axis explained 24.21% of the observed variations, and the second ordination axis explained 17.36%. Among the soil properties, the available K showed the strongest effect, followed by the alkaline-hydrolyzable N and available P. Among the abundance of bacterial genera, *Candidatus* Nitrososphaera showed a certain positive correlation with the alkaline-hydrolyzable N level, and *Nocardioides* was closely correlated with the soil’s available K content. Other bacterial genera, such as *Sphingomonas*, *Candidatus* Solibacter, *Haliangium*, and *Pseudarthrobacter* exhibited a correlation with soil pH, which played a key role in the bacterial community distribution.

## 4 Discussion

### 4.1 Effect of the PSB strains screened from the rhizosphere of carrot

Phosphorus is the second most important macronutrient necessary for plants, after N. P compounds are involved in various physiological and biochemical reactions (Timofeeva et al., 2022). PSB are found in most soils and are estimated to account for 40% of the culturable microbial population (Bakki et al., 2024). In this study, the majority of the microorganisms isolated from the rhizosphere of the carrot were capable of phosphorus-solubilizing ability, indicating the importance of rhizosphere PSB for the growth of carrots. 12 PSB strains isolated in this study belonged to the genera of *Acinetobacter* (7 strains), *Pseudomonas* (3 strains), and *Bacillus* (2 strains). Both *Pseudomonas* and *Bacillus*, recognized as well-documented PGPR, have been reported to be powerful phosphate solubilizers (Ullah et al., 2022; Bouizgarne et al., 2023; Janati et al., 2023; Bakki et al., 2024). The beneficial effects of *Pseudomonas* species, including phosphate solubilization, phytohormones production, and the biosynthesis of antifungal and antibacterial agents, positively influence plant size or fruit yield (Yahya et al., 2021; Wang Z. et al., 2022). Strains of *Bacillus* could stimulate plant growth promotion through the production of auxins or by enhancing the availability and uptake of P (Wang Z. et al., 2022; Bakki et al., 2024). Recently, many studies on PGPR have uncovered that certain PSB strains belong to *Acinetobacter*, such as *A. calcoaceticus* (Ren et al., 2013), *A. rhizosphaerae* (Gulati et al., 2009), and *A. pittii* (He and Wan, 2021; Zhao et al., 2023). The soil sequencing in this study also revealed that the most abundant microorganisms in carrot soil samples belonged to the phylum of Acinetobacteriota (Figure 5a). The phosphate solubilization ability of PSB has been approved to improve seed germination of carrot (Fiodor et al., 2023), and enhance salinity stress during carrot growth (Yadav et al., 2021).

The release of insoluble phosphates in the soil is slow and is regulated by several factors, particularly the soil pH (Devau et al., 2009). Most PSB strains produce indole-3-acetic acid (IAA) and low molecular weight organic acids, creating an acidic pH, which is essential for phosphate solubilization (Patel et al., 2011; Alori et al., 2017). Based on the liquid P dissolution experiment, it was found that for most PBS strains selected in this study, the pH of the medium dropped to the lowest on the 2nd day of cultivation, and the highest P solubilization effect was achieved on the 4th day (Figure 1b). Subsequently, the dissolved P was metabolized by the strain, resulting in an increase in pH and a decrease in the available P content (Figure 1b). Therefore, this feature makes it possible to combine PSB with mineral and organic P, which can increase the efficiency of chemical fertilizers (Duarah et al., 2011; Chen Y. et al., 2021). In addition, PSB can also enhance the efficiency of N fixation (Gupta et al., 2021; Timofeeva et al., 2022), which provides a strategy for the combined use of different functional strains, as well as the co-application of microbial and chemical fertilizers.

Additionally, it is worth noticing that the distribution of PSB has an obvious rhizosphere effect, which means the number and activity of PSB in rhizosphere soils were much higher than those in non-rhizosphere soils (Patel et al., 2011; Li et al., 2019). Also, the phosphatase activity was found much higher in rhizosphere soils than in non-rhizosphere soils (Pan and Cai, 2023). Furthermore, PSB can alter the soil microbial community to increase the P content and related enzyme activity in the soil. This rhizosphere effect is crucial for the construction of PSB communities, which in turn significantly impacts soil processes and enhances crop growth and yield (Pan and Cai, 2023).

### 4.2 Effect of the NFB strain screened from the rhizosphere of carrot

In this study, a rapidly growing strain *B. firmus* MN3 was identified from four NFBs, consistent with previous research that demonstrated the difficulty of isolating NFB from the rhizosphere of non-leguminous plants (Lodeiro, 2015). Studies have shown that NFB strains not only significantly increase plant yields and improve soil fertility, but also affect soil microbial community structure (Stajković-Srbinović et al., 2017). The natural rhizospheric association of *Klebsiella terrigena* E6 and *B. firmus* E3 was reported to have fixed active molecular nitrogen at an effective rate (Zlotnikov et al., 2007). Genome analysis demonstrated that *B. firmus* strain TNAU1 harbors multiple genes related to N fixation (Settu et al., 2024). It has been found that N absorption by the plant could promote the simultaneous absorption of P and K, thus promoting seedling growth (Shi et al., 2023). Also, previously reported effects of strain *B. firmus* SW5 was shown to enhance the uptake of P and K in soybean plants under saline stress (El-Esawi et al., 2018). Similarly, the single use of MN3 strain in this pot experiment was also found to significantly increase the content of soil-available alkaline-hydrolyzable N and P (Figure 3b). Meanwhile, it showed excellent traits in improving the carrot’s root length and plant height (Figure 3a). Moreover, *B. firmus* has shown lethal effects against nematodes, as the toxins it produces can inhibit the hatching of nematode eggs (Ghahremani et al., 2020; Settu et al., 2024). This is significant for root vegetables like carrots, and it also had certain improvements in the morphology of the carrots when used in the combined microbial consortium N3P41K9 in this field trial (Figure 4a).

### 4.3 Effect of the KSB strain screened from the rhizosphere of carrot

Two KSB strains were identified in this study, *B. subtilis* PK9 and *P. polymyxa* MK31. *B. subtilis* is an important biological inoculant with multiple biological functions and multi-stress tolerance (Mehmood et al., 2021). For example, *B. subtilis* PM32 possesses several traits including IAA, phosphate, zinc, and K solubilization, N fixation, siderophore, and extracellular enzyme production, making it a potential biocontrol agent (Mehmood et al., 2021). Other *Bacillus*, such as *B. mucilaginosus* AS1153 (Liu et al., 2017), and *B. amyloliquefaciens* HSE-12 (Li et al., 2023), have also been found to have potassium-solubilizing abilities and genes related to potassium-solubilizing have been studied in *B. aryabhattai* SK1-7as well (Chen Y. et al., 2021). However, *P. polymyxa* has been relatively less studied, but several strains have been proven to have potassium-solubilizing activity as well as the ability to control nematodes (El-Hadad et al., 2011; Sherpa et al., 2021). An increasing number of studies are exploring the possibility of KBS as a substitute for chemical K fertilizers (Anjanadevi et al., 2015). In this study, *B. subtilis* PK9 has a slightly better potassium-solubilizing effect than *P. polymyxa* MK31 (Figure 1c). Furthermore, PK9 showed a significant promoting effect on the root length and weight of carrots, compared to MN3 and MP41 (Figure 3a). This suggested that the PK9 strain served as an excellent PGPR. In the pot experiment, inoculation of PK9 did not enhance the available K in the soil compared to the control group (Figure 3b). It was previously reported the enhancement of plant K nutrition might be due to the stimulation of root growth or the elongation of root hairs by bacteria, Thus, no direct increase in the availability of soil solution K is expected (Meena et al., 2014).

### 4.4 Effect of the combined consortium N3P41K9

Considering the distinct advantages of the strains MN3, MP41, and PK9 in different functional aspects, we attempted to construct a microbial consortium to research the synergistic effect of the combined strains during carrot growth. There is a mutual promotion effect between bacteria with different characteristics among the heterogeneous microbes. For example, PSB can promote biological N fixation, production of phytohormones and various activities related to bio-control (Gupta et al., 2021; Timofeeva et al., 2022). Biofertilizers that contain NFB, PSB and KSB, have high potential for increasing the yield of crops and vegetables (Leaungvutiviroj et al., 2010; Kaur et al., 2024). Therefore, commercial microbial inoculants increasingly adopt the combination of multiple microbial cultures as a consortium, such as WZF, BSW, and PGA used in this study. Moreover, the PGPR can effectively control root-knot nematodes, which is particularly crucial for root crops like carrots. In this study, the constructed consortium N3P41K9 showed better effects on carrot root length and weight as well as soil properties (available chemical elements content and activities of phosphatase, urease, and sucrase) than individual strains (Figures 3, 4), demonstrating additive effects, and its performance is comparable to, or even better than, the microbial fertilizers used commonly in practice. It was previously reported that microbial inoculants had a significant positive correlation with soil enzyme activities, which are involved in most of the biochemical reactions and beneficial to the stability of soil micro-ecosystem (Akhtar et al., 2018; An et al., 2022). Also, it was discovered that the carrot yield and soil properties in the treatment of OPT+N3P41K9 were significantly higher than in the other treatments when chemical fertilizer (OPT) and the best strains of bacteria N3P41K9 were applied simultaneously (Figure 4). This indicates that this treatment had the best effect on N, P, and K uptake and growth promotion of carrot roots.

Although a single inoculation event can enhance crop yields or improve soil properties, establishing long-term colonization of exogenous bacteria in the *in situ* soil remains a challenge (van Elsas et al., 2012). However, the advancement in microbial sequencing technology has enabled us to observe the changes in the soil microbial communities caused by exogenous microbial inoculation. It is worth mentioning that the microbial inoculant of N3P41K9 caused the least disturbance to the indigenous soil microbial community than the chemical fertilizer OPT or the WZF (Figure 5). The stability of soil microbial community diversity can be used to some extent as an indicator of soil ecosystem stability and health. Compared to the CK group, all other groups treated with microbial fertilizer, chemical fertilizer, or a combination of both, exhibited a decrease in final soil pH values. RDA at the genus level indicated that the pH and alkali-hydrolyzable N content played a key role in the bacterial community distribution. Concurrently, the abundance of the acidophilic ammonia-oxidizing archaea (AOA) *Candidatus* Nitrosotalea increased in all groups. AOA is believed to drive nitrification, with ammonia oxidation being the rate-limiting step of this process (Lehtovirta-Morley et al., 2014). AOA predominates in soils, exceeding bacteria by significant amounts, yet their physiology remains largely unknown due to the scarcity of cultivated strains (Lehtovirta-Morley et al., 2011; Lehtovirta-Morley et al., 2014; Herbold et al., 2017). pH and anoxia appear to be key factors regulating AOA community composition in soil (Herrmann et al., 2012). Furthermore, the increase in the abundance of this genus caused by the chemical fertilizer was significantly greater than that caused by microbial fertilizers (Figure 5b), indicating that the application of chemical fertilizer could significantly promote nitrification, which may lead to substantial fertilizer loss and atmospheric and groundwater pollution (Lehtovirta-Morley et al., 2011). Additionally, the beneficial bacterial genus *Gemmatimonas* was also significantly enriched in the field trial of the N3P41K9 group than other groups (Figure 5b). *Gemmatimonas* belongs to the phylum Gemmatimonadetes, which is known as one of the essential genera in phosphate dissolution (Lu et al., 2020). However, in the group OPT+N3P41K9, the increase in the abundance of this genus was minimal, indicating that the use of chemical fertilizer suppressed the enrichment of the PSB. Nocardioide was closely associated with available K content by the RDA, which may be due to the characteristic of Nocardioide that can withstand various low-nutrient conditions and concurrently degrade pollutants (Ma et al., 2023).

## 5 Conclusion

In this study, we investigated the impact of specific PGPR strains or consortium on carrot growth and its rhizosphere microbial community. However, unlike the traditional approach of directly using commercial microbial agents, we isolated multiple indigenous PGPR strains from the carrot rhizosphere and identified three highly efficient strains: MP41, MN3, and PK9, based on their exceptional abilities in P solubilization, N fixation, and K solubilization. These strains were strategically combined to form the microbial consortium N3P41K9, which exceeded the effects of single-strain inoculations in promoting carrot growth and amending soil quality in the pot experiment. Subsequent field trials confirmed the impact of the N3P41K9 consortium, resulting in a significant increase in carrot yields, and improved soil properties, comparable to the performance of commercial microbial fertilizers. Crucially, these benefits were achieved with minimal disruption to the soil’s indigenous microbial community, while also enriching beneficial microbial populations. In addition, this study compared the fertilization practices commonly used by farmers, including chemical fertilizers and microbial fertilizers. The results highlight the potential of microbial consortium N3P41K9 as a beneficial microbial inoculant for the carrot rhizosphere, providing a sustainable alternative to reduce the use of chemical fertilizers. This approach can also be extended to other root crops with the aim of creating a network of PGPR, which will promote the development of demand-based sustainable agriculture.

## Data Availability

The data presented in this study are deposited in the NCBI repository, accession number PRJNA1161228. Further inquiries can be directed to the corresponding author.

## Footnotes

1
https://itol.embl.de/
