# Supplementary material for: Enhancing carrot (Daucus carota var. sativa Hoffm.) plant productivity with combined rhizosphere microbial consortium
Source: Front Microbiol. 2024 Nov 20;15:1466300. doi: 10.3389/fmicb.2024.1466300 (PMC11615968; doi:10.3389/fmicb.2024.1466300)
Supplement: Supplementary file 2 [file Image_1.pdf]

Supplemental

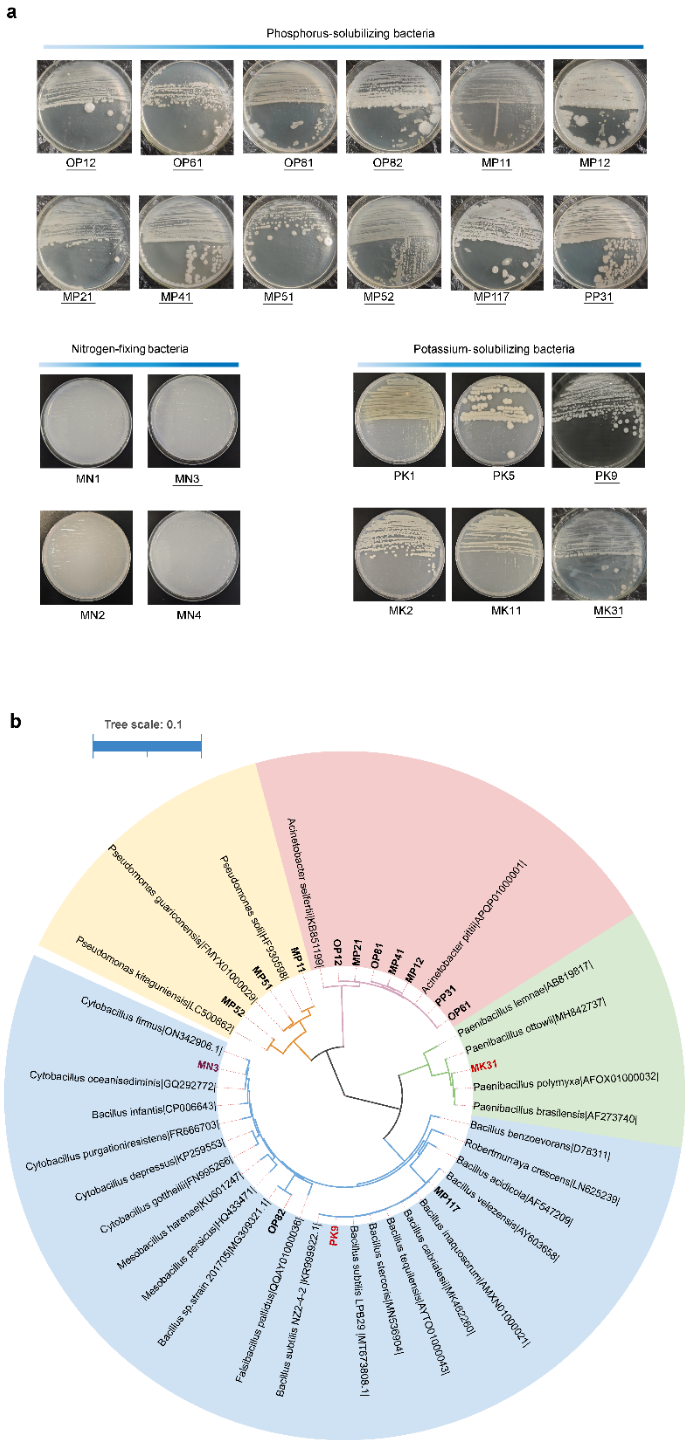

Supplementary Figure 1. The morphology and phylogenetic tree analysis of the bacterial strains. (a) The plate culture morphology of partial of the screened phosphate-solubilizing bacteria, nitrogen-fixing bacteria, and potassium-solubilizing bacteria. The selected strains used in this study are underlined. (b) Phylogenetic tree analysis of the identified strains. The different background colors indicate different genera. The nitrogen-fixing bacteria MN3 and Potassium-solubilizing bacteria MK31 and PK9 are shown in red.

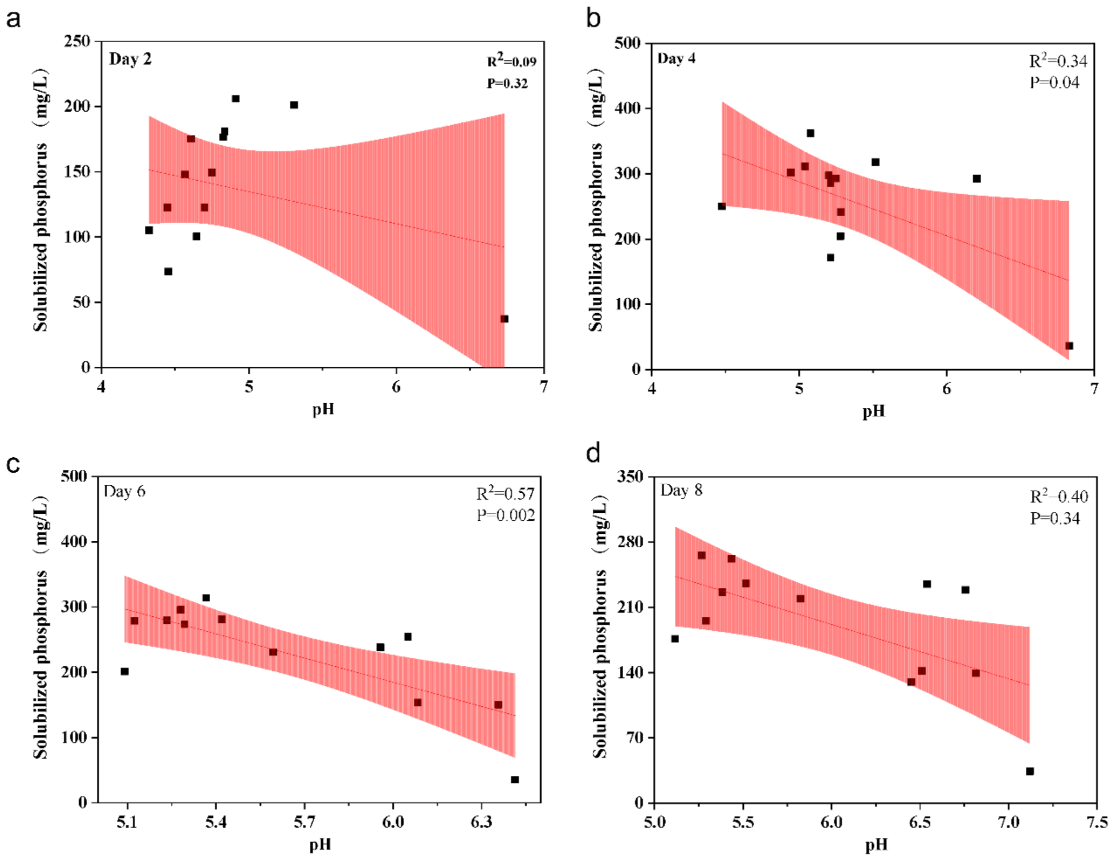

Supplementary Figure 2. Linear regression analysis between pH and phosphate soluble content on Day 2 (a), Day 4 (b), Day 6 (c), and Day 8 (d). The horizontal coordinate indicates pH value and the vertical coordinate indicates the amount of solubilized phosphorus.

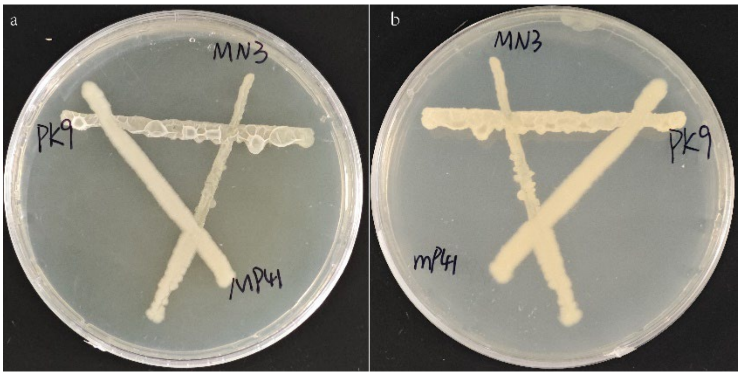

Supplementary Figure 3. Verification of strain growth antagonism. View from the top (a) and the bottom (b) of the cultural plate. The three strains used are the NFB strain MN3, PSB strain MP41, and KSB strain KP9.

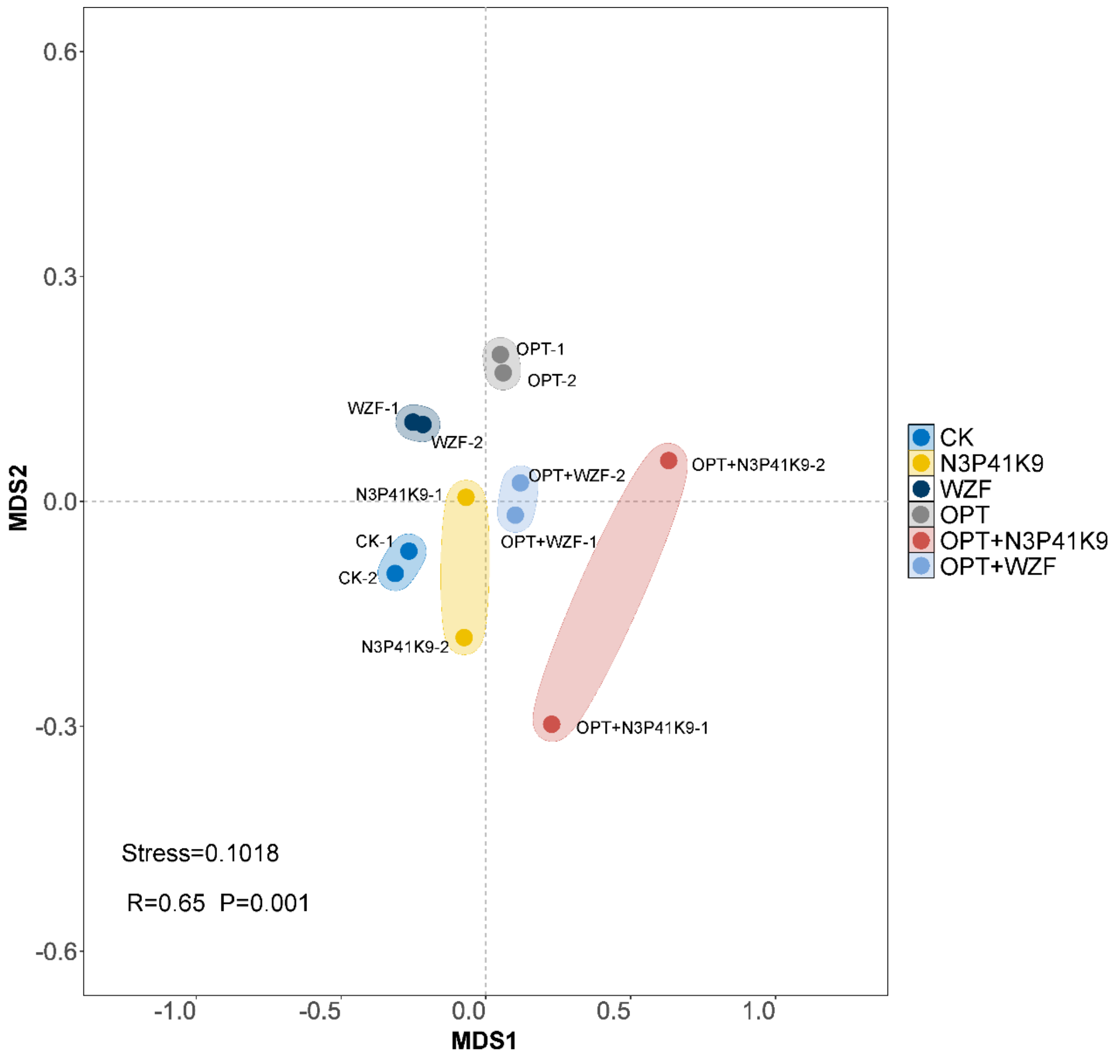

Supplementary Figure 4. The differences in rhizosphere microbial community composition among six groups. The NMDS plot, generated using the Weighted UniFrac distance and the Clark method, illustrates significant differences in bacterial community composition across the six groups, encompassed within a 95% confidence interval. ANOSIM confirms further the significant difference (R = 0.65, p = 0.001).
